# Post‐COVID‐19 fatal *Aspergillus* endocarditis: A case report

**DOI:** 10.1002/jcla.24816

**Published:** 2022-12-19

**Authors:** Narges Najafi, Azam Moslemi, Rahman Ghafari, Shadi Shayesteh Azar, Maryam Nabati, Leila Faeli, Maryam Salimi, Roghayeh Mirzakhani, Tahereh Shokohi

**Affiliations:** ^1^ Department of Infectious Diseases, Antimicrobial Resistance Research Center, Communicable Diseases Institute Mazandaran University of Medical Sciences Ghaemshahr Iran; ^2^ Student Research Committee Mazandaran University of Medical Sciences Sari Iran; ^3^ Department of Cardiac Surgery, Cardiovascular Research Center, Mazandaran Heart Center Mazandaran University of Medical Sciences Sari Iran; ^4^ Department of Cardiology, Faculty of Medicine Cardiovascular Research Center Mazandaran University of Medical Sciences Sari Iran; ^5^ Mazandaran Heart Center Mazandaran University of Medical Sciences Sari Iran; ^6^ Invasive Fungi Research Center, Communicable Diseases Institute Mazandaran University of Medical Sciences Sari Iran; ^7^ Department of Medical Mycology, School of Medicine Mazandaran University of Medical Sciences Sari Iran

**Keywords:** *Aspergillus fumigatus*, COVID‐19, endocarditis, prosthetic heart valves

## Abstract

**Background:**

*Aspergillus* endocarditis (AE) is a rare fatal infection. The infection is often reported in patients with prosthetic heart valves, immunosuppressed, broad‐spectrum antimicrobial use regimens, and drug abusers.

**Methods:**

Herein, we report a rare case of native mitral valve AE in a 63‐year‐old man, with a probable COVID‐19‐associated invasive pulmonary aspergillosis nine months ago treated with antifungals.

**Results:**

In the last admission, the lethargy, neurological deficit, and septic‐embolic brain abscess in brain MRI led to suspicion of infective endocarditis. Transesophageal two‐dimensional echocardiography and color Doppler flow velocity mapping showed a large highly mobile mass destroying leaflet and severe mitral regurgitation. The Surgical valve replacement is performed. The surgical valve replacement is performed. Direct microscopic examination and culture of the explanted and vegetative mass revealed *Aspergillus* section *Fumiagati* confirmed by molecular method. Despite the administration of voriconazole and transient improvement the patient expired.

**Conclusion:**

As AE is a late consequence of COVID‐19‐associated invasive pulmonary aspergillosis, therefore, long‐term follow‐up of invasive aspergillosis, and prompt diagnosis of surgical and systemic antifungal therapy treatment, are warranted to provide robust management.

## Graphical abstract

Graphical abstract shows a chronological summary of the patient's manifestation, laboratory diagnosis, and treatment from the onset of COVID‐19 pneumonia‐related symptoms to the development of the *Aspergillus* endocarditis.

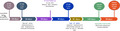

## 1 INTRODUCTION

*Aspergillus* endocarditis (AE) has been reported in immunocompromised and immunocompetent patients with a history of open‐heart surgery, valve replacement, or intravenous drug use. The high mortality rate among survivors is very important due to the risk of relapsing infection.^1^

A literature review from 2005 to 2016 showed that out of 374 cases of infective endocarditis, 43 (11.5%) had fungal infections, and the most common fungi (31/374 patients; 8.3%) were *Aspergillus* and *Candida* (9/374 patients; 2.4%).^2^ Extracardiac manifestations, mistaken/delayed AE diagnosis, and long duration of symptoms before going to the hospital may contribute to wrong management in patients then causing a delay in the treatment and conclusive poor outcomes.^3^

The SARS‐CoV‐2 virus is a novel coronavirus caused by respiratory tract infection with asymptomatic to severe infection presentation. The most common simultaneity disease found in COVID‐19 patients is cardiovascular disease.^4^, ^5^ Since the onset of the COVID‐19 pandemic, multiple reports of invasive pulmonary aspergillosis have emerged,^6^ but COVID‐19‐associated *Aspergillus* endocarditis is rare. Herein, we report a rare case of native mitral valve AE in a patient with post‐COVID‐19‐associated invasive pulmonary aspergillosis.

## 2 CASE REPORT

On May 13, 2022, a 63‐year‐old man, with severe weakness, lethargy, unilateral paralysis inability to stand up, and reduced force in the right upper limb, was admitted to the Mazandaran Heart Center affiliated with Mazandaran University of Medical Sciences, northern Iran.

His past medical history revealed that he got COVID‐19 about 9 months ago and was admitted to the hospital due to decreased oxygen saturation (SpO_2_) and received remdesivir and methyl‐ prednisolone pulse therapy for 10 days and was discharged in a good condition. He was readmitted to the emergency department with the symptoms of red eyes and blurred vision 2 days later, and a CT scan and ophthalmological consultation highly suggestive of fungal infection, and started the treatment with amphotericin B. The three consecutive serum galactomannan assays showed one positive 6.1 and two negative results. However, despite the treatment, the visual acuity of his right eye decreased during hospitalization. Finally, after day 26 of hospitalization, the patient had been discharged with continuing voriconazole at home.

Two weeks later, the patient's blood test results were as follows: WBC = 6.1 × 10^9^/L, RBC = 3.94 × 10^6^/μl, HB = 11.4 g/dl, ESR = 66(H) mm/h, AST = 94 U/L(H), ALT = 195 U/L(H), D‐DIMER = 1533 ng/ml, CRP = 8 mg/L (H) COVID IgG = 9.8(H), COVID IgM = 0.2.

After 56 days of the first admission in a follow‐up visit with an infectious disease specialist, a CT scan of the lung and mediastinum revealed a bilateral ground‐glass opacity (GGO) in the upper and peripheral areas and a 38 × 28 mm cavitary in the right lower lobe with fibrotic changes in the form of bronchiectasis and an increase in the thickness of the adjacent pleura in the context of possible old infections.

After 3 months of the first admission, he was admitted with complaints of loss of vision to the Department of Ophthalmology, Labbafinejad Medical Center with suspicion of fungal involvement. The necrotic tissues were surgically debrided without exenteration and treated with liposomal amphotericin B, and he was discharged after 1 month of hospitalization with a recommendation to continue oral voriconazole. The voriconazole had been discontinued on her decision after 1 week probably because of its shortage through public health insurance and the high cost in the illegal black market.

**FIGURE 1:**
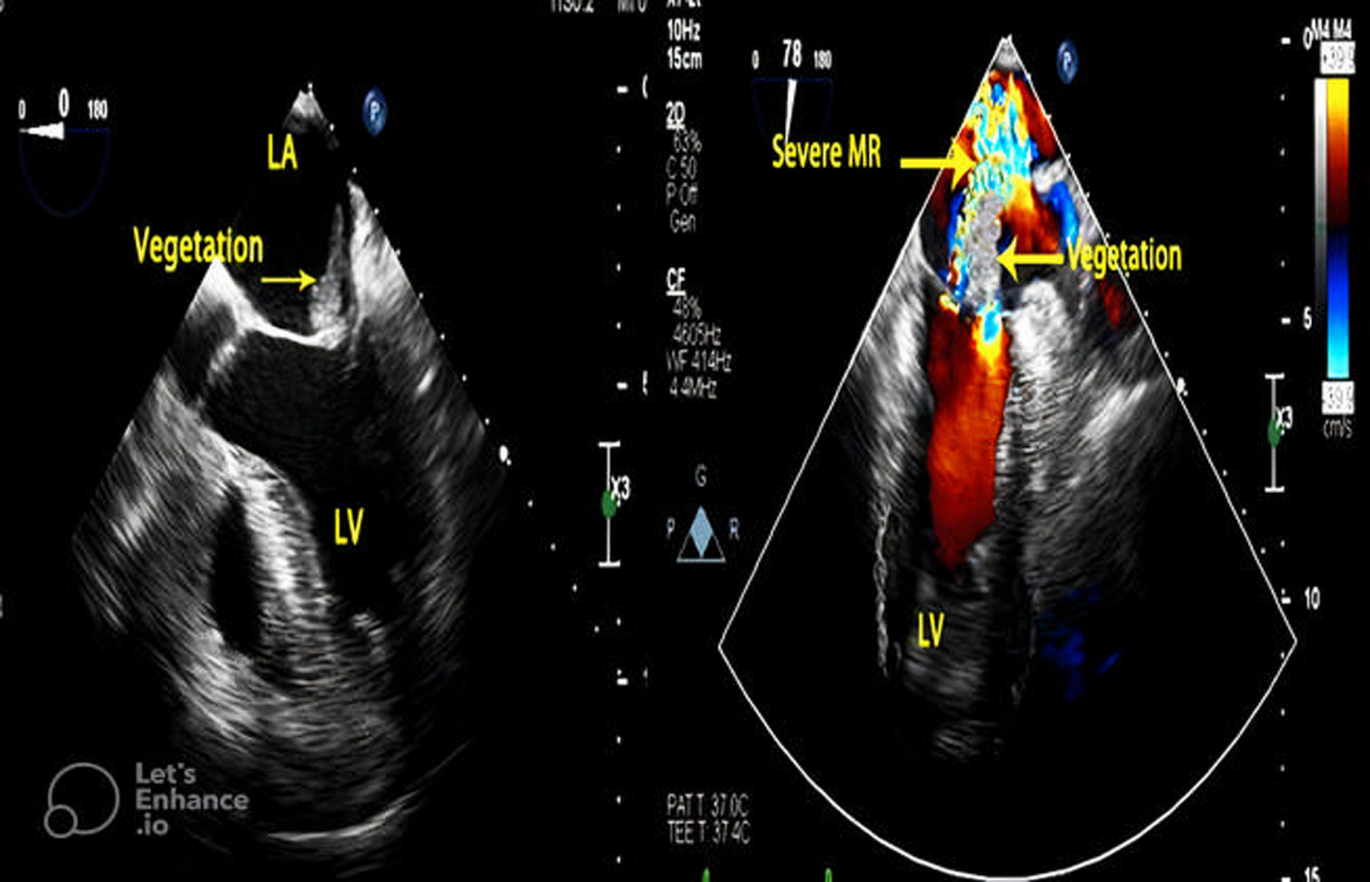
Transesophageal echocardiography in mid‐esophageal view (0 degree) showed large vegetation attached to the atrial side of anterior mitral valve leaflet (A), and Transesophageal two‐dimensional echocardiography and color Doppler flow velocity mapping in the mid‐esophageal long‐axis view (78 degree) revealed severe MR due to infective destruction of the anterior mitral leaflet (B). LA, left atrium; LV, left ventricle; MR, mitral regurgitation

In the patient's last admission to the heart center on May 13, 2022, his laboratory report was as follows: (WBC = 12.9 × 10^9^/L, RBC = 4.57 × 10^6^/μl, HB = 12 g/dl, PLT = 267,000, CRP =155 mg/L, Creatinine = 1.74 mg/dL, BS = 166 mg/dl, LDH = 1362 U/L, Troponin = 2.36 ng/ml, PT = 13.1 s, PTT = 37 s, INR = 1.05, D‐Dimer = 1.89 μg/ml). Due to exacerbation weakness and lethargy and neurological deficit, brain MRI and neurological consultation were ordered which led to a diagnosis of septic‐embolic brain abscess possibly complicated by infective endocarditis. Transthoracic echocardiographic showed normal left ventricular size with mild systolic dysfunction (LVEF = 45%–50%). Transesophageal two‐dimensional echocardiography and color Doppler flow velocity mapping showed a large highly mobile mass (3.1 × 1.5 cm) on the atrial side of the anterior mitral valve leaflet highly suggestive of infective fungal vegetation resulting in a destructing leaflet and severe eccentric mitral regurgitation. Also, there was mild tricuspid regurgitation and high normal pulmonary arterial pressure (pulmonary artery pressure = 35 mmHg). These findings were highly suggestive of mitral valve infective endocarditis (Figure 1).

The cardiac surgery team recommended mitral valve replacement. However, the surgery was delayed because of the patient's altered mental status in which he had drowsiness and confusion and also for the treatment of cerebral embolism. In the meantime, the patient underwent antibiotic therapy under the supervision of an infectious disease specialist with tazocin, vancomycin, and amphotericin B.

**FIGURE 2:**
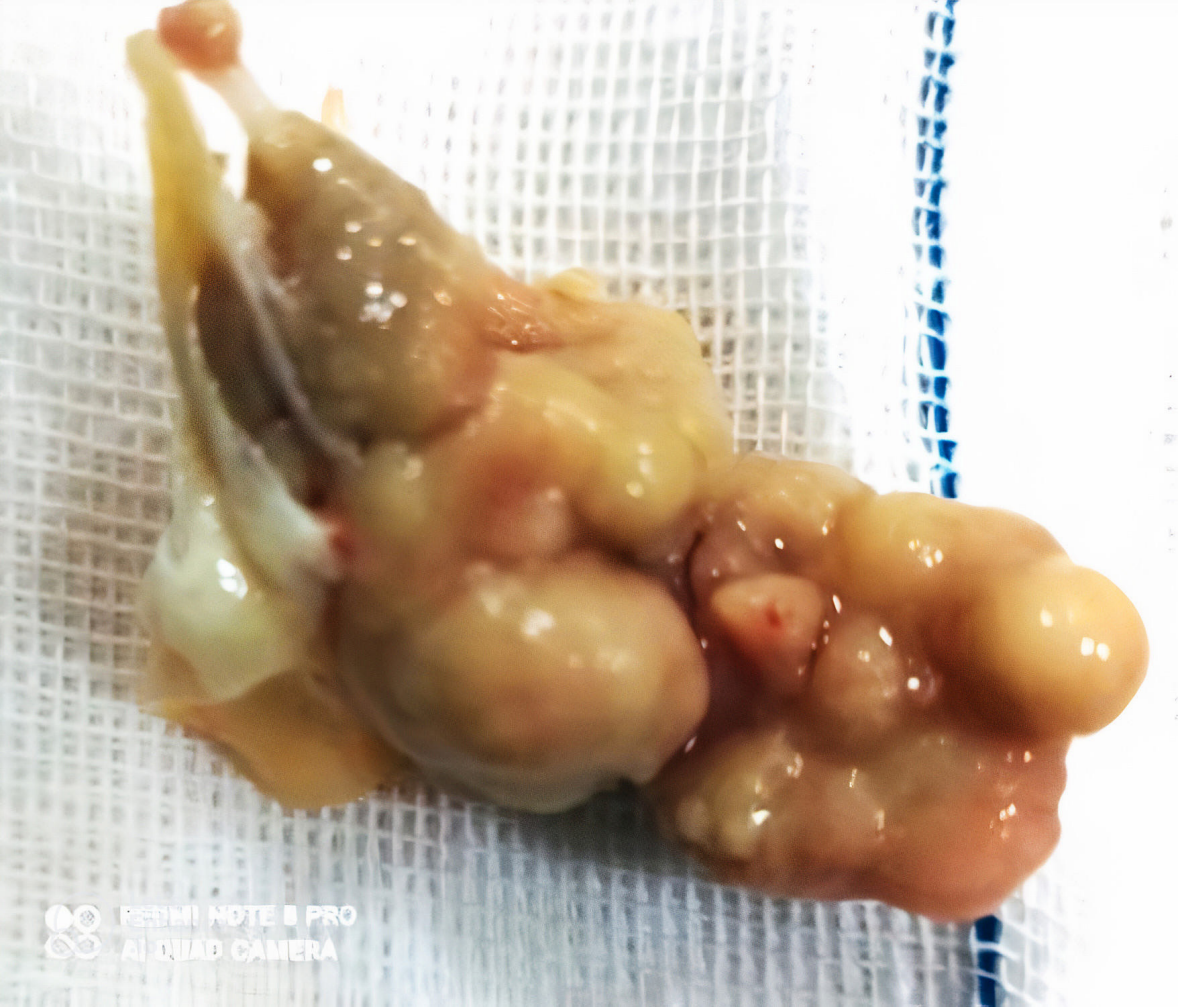
Explanted mitral valve vegetation

After consultation with a neurologist, the infected mitral valve was removed and replaced by the prosthetic mechanical valve successfully, and the use of warfarin was recommended. The explanted valve and vegetation (Figure 2) were sent to the reference Mycology Laboratory.

**FIGURE 3:**
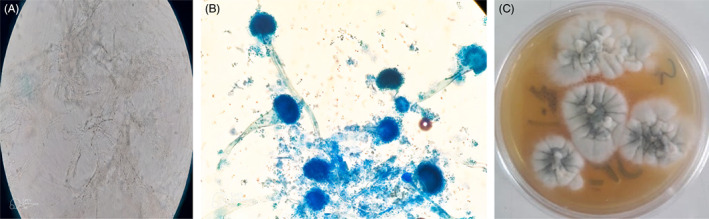
(A) Dichotomous branching hyphae in KOH preparation (40× magnification). (B) Bluish‐green *Aspergillus* colonies on Sabouraud dextrose agar. (C) The microscopic appearance of *Aspergillus fumigatus* shows uniseriate phialides with columnar conidial heads and flask‐shaped vesicles.

Direct microscopic examination of the KOH mount of the tissue showed a mass of dichotomous branching hyphae (Figure 3A). The culture of biopsied tissue onto Sabouraud dextrose agar for 1‐day incubation at 27°C yielded *Aspergillus* species (Figure 3B). The microscopic evaluation of the grown *Aspergillus* colonies showed uniseriate phialides with columnar conidial heads and flask‐shaped vesicles (Figure 3C). Based on microscopic characteristics, the fungus was identified as a member of the *Aspergillus* section *Fumiagati* and confirmed by amplification of the β tubulin gene by polymerase chain reaction. The amplicons were sequenced and align with the GenBank database (https://blast.ncbi.nlm.nih.gov/Blast.cgi) for accurate identification. The obtained sequences were submitted to GenBank and received accession number ON922989.

The antifungal susceptibility test of the isolate was performed according to the Clinical and Laboratories Standards Institute protocol (CLSI M38‐A3).^7^ The minimum inhibitory concentration (MIC) values for itraconazole; 0.5 μg/ml, voriconazole; 0.25 μg/ml, amphotericin B; 0.25 μg/ml, posaconazole; 8 μg/ml, ravuconazole; 8 μg/ml, MEC values for caspofungin, and anidulafungin both; >32 μg/ml were determined. So, the prescription of voriconazole was continued.

The patient was in good general condition and improved during the hospitalization, but he suddenly lost consciousness. A left intracerebral hemorrhage (ICH) was proved in the examination. Despite efforts to save the patient's life, the patient died after 29 days after the last admission.

The Ethics Committee of Mazandaran University of Medical Sciences, Sari, Iran approved this report (IR.MAZUMS.REC.1399.551), and informed consent was obtained from the legal guardian for its details to be included in the manuscript and for publication.

## 3 DISCUSSION

Severe inflammatory responses, which are in the form of cytokine storms, are seen in patients with COVID‐19.^8^ We can assume that increased inflammation during this COVID‐19 disease, damages the heart valves and creates a suitable surface, thus binding fungi or other pathogens to the heart valve and causing infective endocarditis.

Over the past year, with the increasing number of patients with COVID‐19, cases of COVID‐19 patients presenting with infective endocarditis have increased.^9^ A study in 2021 suggested that the SARS‐CoV‐2 virus pandemic has outlined a new problem for the diagnosis of cardiovascular diseases and further care and treatment.^4^

A review of endocarditis after COVID‐19 showed that the highest percentage of infective endocarditis was related to the mitral valve (33%) of the heart,^8^ which is similar to our finding. Patients, who do not have surgery on an infected valve, pose a 100% risk of mortality while in patients who undergo cardiac valve replacement, 45% is reported.^1^

Despite appropriate treatment measures, infective endocarditis has a high morbidity and mortality rate. Mortality rates can be approximately 25%.^10^

Our report addressed the criteria of probable invasive pulmonary aspergillosis presented with a bilateral ground‐glass opacity, cavitation, and bronchiectasis 9 months before the diagnosis of AE. The immunocompromising condition was steroid therapy, direct damage of respiratory virus to airway epithelium, and lymphopenia in the context of ARDS associated with COVID‐19. In general, *Aspergillus fumigatus* has been reported to be the most common cause of endocarditis and invasive infections,^1^ which has also been the cause of infection in our case report.

In a patient with heart failure, surgery is the best option, and the management of infective endocarditis (IE) may be difficult, as there are several limitations to normal practice in the era of the COVID‐19 pandemic.^5^ Our report illustrates the challenges in the standard management of the disease and the potential severity of complications, which remains unknown about COVID‐19 management in special populations.

There is a limited report on COVID‐19 and IE. The authors raised a hypothesis that systemic inflammation caused by SARS‐CoV‐2 is the potential risk factor for IE and has devastating prothrombotic effects.^11^ The vegetation developed through transient bacteremia and attachment to damaged vulvar endothelium associated with the COVID‐19 virus.^12^

Hospital‐acquired infective endocarditis (HAIE) is a life‐threatening complication of medical procedures. The risk of HAIE may add to the prolongation of the ICU stay as a potential risk factor during an outbreak of coronavirus infection.^13^

In our case, the treatment of probable IPA involves voriconazole that is active in vitro against *Aspergillus fumigatus* isolated species for 9 months. It is worth noting that the patient often stopped taking the medication without a consultant and had irregular follow‐up visits that may be a result of COVID‐19 visiting restriction and/or antifungal supply led to fatal consequences. Many studies show the incidence of infective endocarditis (IE) remains high, between 1.5 and 9.6 cases per 100,000, and the mortality rate is about 25%, despite advances in medical care.^14^

## 4 CONCLUSION

Among the reported cases of *Aspergillus* endocarditis, we report a novel case of *Aspergillus* endocarditis after getting infected with COVID‐19. The management and treatment of *Aspergillus* endocarditis may be difficult since there are several limitations to normal practice during this pandemic of COVID‐19. This case illustrates the possible seriousness of complications and the challenges in developing standard diagnosis and management. The present report highlights the occurrence of AE as a late consequence of probable COVID‐19‐associated pulmonary aspergillosis. It should bring the attention of physicians toward an extended follow‐up visit in a patient with COVID‐19 for probable fungal infection and cardiovascular monitoring, early diagnosis, and timely surgical and antifungal treatments are warranted to provide robust management.

## AUTHOR CONTRIBUTIONS

All authors have made substantial contributions to the conception and acquisition of data, drafting of the article, and approval of the final version.

## FUNDING INFORMATION

The author would like to thank the Invasive Fungi Research Center of Mazandaran University of Medical Sciences, Sari, Iran for financial support (grant no. 1327).

## CONFLICT OF INTEREST

None.

## Data Availability

No data are available
